# A Practical Treatise on Fractures and Dislocations

**Published:** 1860-05

**Authors:** 


A Practical Treatise on Fractures and Dislocations. By Frank Hastings Hamilton, Prof. of Surgery in the University of Buffalo, etc. Blanchard & Lea, Philadelphia.

This is almost the only modern work in the English language which can claim the title of a systematic and full treatise upon dislocations and fractures. Its author is already favorably known in the professional literature of this country. His Fracture Tables, published some years since, giving laborious statistics on the subject of fractures and their results, probably furnish effectual defence to more cases of malpractice persecution than anything that has ever been printed. Indeed the mania for malpractice suits visibly diminished from the time that Dr. Hamilton's work made its appearance in the courts.

The present effort is not statistical, but is intended for a systematic treatise. About five hundred pages are devoted to fractures, their causes, symptoms, treatment, and the apparatus therefor. Two hundred and fifty pages are then assigned to the various dislocations. The whole is illustrated by two hundred and eighty-nine wood cuts.

The illustrations of different kinds of apparatus and dressings are very numerous and valuable, and the clear description of the methods employed are of the utmost use to any one who desires to familiarize himself with this subject.

A work like this is in some respects more useful than translations of foreign treatises, because it gives the latest results of American ingenuity, and practical applications of surgical principles. The American branch of the profession is less profound in thought than the German, and less laborious in pathological research than the French; but it is probably superior to the world in its capacity for applying principles to practice. Hence, some of the finest advances in practical surgery are of American origin, while the great discoveries in principles and in special pathology nearly all come from Europe.

The work before us is eminently American, in that it is essentially practical, and as such we recommend it to our brethren of the surgical profession.

